# SAFE: A Novel Microwave Imaging System Design for Breast Cancer Screening and Early Detection—Clinical Evaluation

**DOI:** 10.3390/diagnostics11030533

**Published:** 2021-03-16

**Authors:** Aleksandar Janjic, Mehmet Cayoren, Ibrahim Akduman, Tuba Yilmaz, Emre Onemli, Onur Bugdayci, Mustafa Erkin Aribal

**Affiliations:** 1 Mitos Medical Technologies, ITU Ayazaga Ari Teknokent 2-B Block 2-2-E, Maslak, 34469 Istanbul, Turkey; cayoren@itu.edu.tr (M.C.); akduman@itu.edu.tr (I.A.); tuba.yilmaz@itu.edu.tr (T.Y.); onemli17@itu.edu.tr (E.O.); erkin.aribal@acibadem.com (M.E.A.); 2 Electrical and Electronics Engineering Faculty, Istanbul Technical University, Maslak, 34469 Istanbul, Turkey; 3 Department of Radiology, School of Medicine, Marmara University, Pendik, 34899 Istanbul, Turkey; onur.bugdayci@marmara.edu.tr; 4 Radiology Department, Breast Health Center, Altunizade Hospital, Acibadem M.A.A. University, Atasehir, 34684 Istanbul, Turkey

**Keywords:** breast cancer, microwave imaging, early diagnosis, clinical study

## Abstract

SAFE (Scan and Find Early) is a novel microwave imaging device intended for breast cancer screening and early detection. SAFE is based on the use of harmless electromagnetic waves and can provide relevant initial diagnostic information without resorting to X-rays. Because of SAFE’s harmless effect on organic tissue, imaging can be performed repeatedly. In addition, the scanning process itself is not painful since breast compression is not required. Because of the absence of physical compression, SAFE can also detect tumors that are close to the thoracic wall. A total number of 115 patients underwent the SAFE scanning procedure, and the resultant images were compared with available magnetic resonance (MR), ultrasound, and mammography images in order to determine the correct detection rate. A sensitivity of 63% was achieved. Breast size influenced overall sensitivity, as sensitivity was lower in smaller breasts (51%) compared to larger ones (74%). Even though this is only a preliminary study, the results show promising concordance with clinical reports, thus encouraging further SAFE clinical studies.

## 1. Introduction

Breast cancer accounts for approximately 25% of the total number of cancers diagnosed in women worldwide, with around 2 million new cases arising every year [1,2,3]. The American Cancer Society reports that 5-year breast cancer survival rates for localized and locally advanced cases are 99% and 86%, respectively, showing that early detection is associated with lower breast cancer mortality [3,4,5].

Nowadays, mammography is widely available and used for breast cancer screening and diagnosis. It is reported that, in the US, mammography screening has contributed to a 40% reduction in breast cancer-associated mortality between 1989–2016 [6]. However, there are also opposing views. In their systematic review, Gøtzsche and Jørgensen reported that randomized control studies of sufficient quality did not show any evidence of a benefit from mammography screening [7]. Mammography itself is limited by several known risks such as overdiagnosis, overtreatment, false-positive examinations, and radiation exposure [8]. Additional limitations such as relatively low sensitivity, patient discomfort, and pain during the procedure due to breast compression and false negativity in dense breasts [9,10,11,12,13] also impact mammography screening reliability. Screening mammography is discouraged in women with an average risk under 40 years of age because of increased breast density in younger women and cumulative radiation dose over the years. Ionizing radiation also limits repeated examinations.

In order to surpass the aforementioned limitations, a significant number of studies related to the development of alternative imaging methods based on the implementation of microwave technology for breast cancer imaging have been conducted [14,15,16,17]. Microwave imaging (MWI) represents an imaging modality by which the unknown attributes (location, shape, or electromagnetic properties) of the objects-of-interest (OI) are reconstructed based on the measured electromagnetic field OI scatters. The OI is located inside the imaging domain and is illuminated by the number of non-ionizing radiation sources. For each source employed, the scattered field, which arises because of a difference between the electromagnetic properties of OI and the surrounding “background”, is measured, and the measured data are inverted by an imaging algorithm in order to acquire the resulting image. In that sense, the MWI approach for breast cancer screening and early detection relies on exploiting the difference in the electromagnetic properties of healthy and cancerous breast tissue. Previous studies have shown that cancerous breast tissue is characterized by a higher dielectric permittivity compared to healthy tissue [18,19,20,21,22,23,24,25]. This permittivity difference can be explained by the fact that cancers are characterized by an increased level of fluid or increased vascularity within the tumor compared to healthy tissue.

So far, seven MWI prototypes were clinically tested, where only two of them, a system developed at Dartmouth College, USA (DC) and the Multistatic Array Processing for Radiowave Image Acquisition (MARIA^®^) system developed at the University of Bristol, UK, were studied on significant number of patients [26,27]. Motivated by the lack of comprehensive MWI clinical assessments (in terms of the number of patients involved) and our desire to improve the area of breast cancer imaging, we developed a device for screening and early cancer diagnosis, namely SAFE (Scan and Find Early), which is based on non-ionizing microwave radiation. The method does not require breast compression, providing a painless scanning procedure for patients.

Beside MWI, there are several ongoing studies considering alternative imaging methodologies based on non-ionizing radiation and without the need for breast compression, such as optical tomography and ultrasound tomography. The preliminary studies show that, although ultrasound tomography can contribute to the diagnosis, there are not sufficient data to support its role in screening [28,29]. Similarly, studies employing optical tomography have shown the value of imaging system in differential diagnosis of breast lesions but have always been used as a supplementary modality to other imaging methods, indicating that it is not yet ready for “standalone” use [30,31]. The current study differs from these modalities, as MW breast imaging can be used in screening rather than differential diagnosis. The ability of SAFE to detect malignant lesions as a screening methodology may add a value in triaging true negative patients, thus reducing the number of unnecessary mammography screenings.

In the following sections, we discuss the complete SAFE protocol as well as of other applied imaging modalities, together with the ways of collecting and evaluating the acquired data. Furthermore, in the Results section, we make an overall evaluation and comparison of SAFE imaging results with the clinical reports provided by the Radiology Department of Marmara University Hospital.

## 2. Materials and Methods

### 2.1. SAFE Clinical Protocol

**Figure 1:**
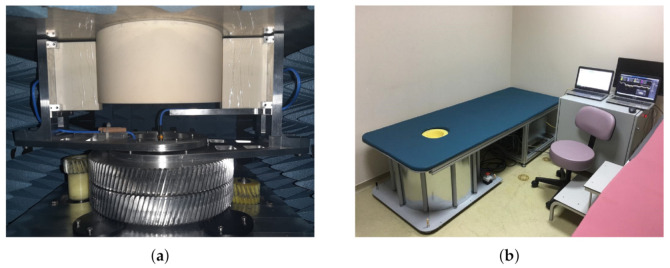
SAFE (Scan and Find Early) scanning configuration and bed system: (**a**) mehanically mobile bistatic system producing 1296 measurements for each frequency employed and (**b**) the SAFE design in a clinical surrounding.

The SAFE study and clinical implementation were approved by the Ethics committee of Marmara University School of Medicine. Patients were enrolled in compliance with Institutional regulations. Patient participation was voluntary after signing a written informed consent. Only patients who were considered for biopsy after routine imaging (mammography, ultrasonography, or MRI) were enrolled in the study. SAFE scanning was performed by the Mitos Medical Technologies medical staff before the biopsy. For each SAFE scanning session, patients were required to lie prone on the table with one breast inserted into the coupling medium cup. The cup itself is a part of the coupling cylinder, which is required for impedance matching. A mechanically mobile bistatic system was employed, in which transmitting and receiving antennas were touching and circling around the coupling cylinder (Figure 1a). For every transmitter position, 36 receiving points were available, making a total of 1296 (36 transmitting positions) measurements for each frequency employed. The operating frequency band employed was between 1.4 GHz and 8 GHz, chosen to achieve a suitable balance between absorption rate and resolution. The data acquisition step was 200 MHz. Each breast was imaged under the same conditions individually. Since breast size differs between patient, size adjustable cups were made available. The total scanning time was approximately 20 min (15 min for data acquisition of both breasts and additional 5 min for patient positioning). Each image was processed by using a MATLAB graphical user interface, with the processing time of approximately 5 s. Image reconstruction was performed after completion of the scan and was based on the inverse scattering algorithm described in one of the previous related device studies [32].

### 2.2. Mammography

Mammography imaging was performed on a full field digital mammography unit (Mammomat Inspiration, Siemens Healthcare, Erlangen, Germany) in standard two views (craniocaudal and medio-lateral oblique) for each breast.

### 2.3. Ultrasonography

Breast ultrasound imaging and breast biopsies were performed on a Toshiba Aplio 400 scanner (Canon Medical Systems Corporation, Tochigi, Japan) using a 5–14 MHz linear transducer. The side, location, size, and depth of the lesion were recorded during the ultrasound examination.

### 2.4. Magnetic Resonance Imaging

Breast MR images were acquired using a 3 Tesla scanner (Magnetom Verio, Siemens Healthcare, Erlangen, Germany) with the patient in prone position with a 16-channel phased array dedicated breast coil (Siemens Healthcare). Our standard examination protocol included the following sequences: (1) Axial turbo spin-echo fat saturated T2-weighted sequence (TR/TE, 4100/70 ms; field of view, 30 cm; acquisition matrix, 440 × 380; slice thickness, 3 mm); (2) diffusion-weighted imaging using echo-planar image (EPI) sequence with fat suppression (b values 50, 400, and 800 s/mm${}^{2}$; TR/TE 9700/86 ms; FOV, 30 cm; in-plane resolution, 1.7, 2 mm${}^{2}$; slice thickness, 3 mm); and (3) 3D volumetric interpolated (VIBE) sequence (TR/TE, 5.01/1.77 ms; FOV, 30 cm; acquisition matrix, 512 × 460; slice thickness, 1 mm; in-plane resolution, 0.6, 0.7, 1.0 mm) for dynamic contrast-enhanced sequence. For dynamic contrast enhanced imaging, either Gadobutrol 0.1 mmol/kg (Bayer Schering Pharma AG, Leverkusen, Germany) or Gadoterate Meglubine 0.1 mmol/kg (Guerbet) were administered intravenously via an antecubital vein at a rate of 2 mL/s using an automated injector system (Medrad Spectris Solaris EP, Bayer Medical Care, Berlin, Germany) followed by a saline injection. Subtracted contrast enhanced dynamic images were used as a standard for lesion identification.

### 2.5. Data Evaluation

Data collected throughout the SAFE scanning procedure were analyzed by the engineering department and medical staff of Mitos Medical Technologies company (supervised by the Radiology Department of Marmara University Hospital Breast Center) who had extensive training in analyzing numerical, experimental, and clinical microwave imaging data over the course of 4 years. The non-blinded evaluation process aimed to identify the approximate position, size, and shape of the affected region and to compare the results to the radiological evaluation of the patients. Sensitivity was defined as a true positive outcome of the evaluation process. In the imaging phase, we used a differential microwave imaging approach, which requires us to a use a reference measurement to supress the background [33]. At this stage, we chose one of the breast measurement as a reference to obtain a differential image, which revealed possible anomalies in the patient’s breasts. All acquired images were pseudo color images, where colormap intensities of the acquired images correlate with breast tissues dielectric profiles, such that higher dielectric permittivities correspond to higher intensity values. Each colormap intensity value corresponds to its absolute value. None of the postprocessing or image adjustments steps, including colormap modifications, were done during the image reviewing process.

## 3. Results

A total number of 115 patients underwent the SAFE scanning protocol, where the sensitivity for lesion detection was 63% (72/115). Sensitivity for different lesion types was as follows: benign (64% (42/66)), high-risk (B3 lesion) (88% (7/8)), and malignant (59% (24/41)). High-risk or B3 lesions are defined as lesions with uncertain malignant potential. Benign cases were mostly dominated by fibroepithelial lesions, with 24 cases in total (14/24). Beside fibroepithelial lesions, the pathology showed the presence of adenosis (8/10), sclerosing adenosis (1/1), acute and chronic inflammatory changes (5/8), chronic inflammatory changes (2/6), inflammatory changes (1/1), columnar cell changes (3/4), fibrosis (4/4), granulomatous mastitis (0/1), granulomatous lenfadenitis (1/1), ductal ectasia (1/1), pseudoangiomatous hyperplasia (0/1), and fat necrosis (2/2). Two cases were not evaluated histopathologically but were clinically diagnosed as abscess formation. In the high-risk group, intraductal papilloma was the most common type of lesion, with 5 cases, where all of them were detected correctly. In addition, the presence of flat epithelial atypia (1/1), atypia (1/1), and intruductal papilloma with atypia (0/1) was noticed. Invasive ductal carcinoma (IDC) was the most dominant type of malignancy (17/30). Besides IDC, invasive lobular carcinoma (2/4), ductal carcinoma in situ (3/5), papillary malignancy (1/1), and lymphoma (1/1) were present. Two cases of bilateral disease were present, but our device could not detect either of them.

Sensitivity was lower in smaller breasts (51% (28/55)) compared to larger size breasts (74% (44/60)). Patient age ranged from 18 to 86 years (average 46 years). The smallest detected lesion size was 6 mm, whereas the mass size ranged from 6 mm up to the 120 mm. The average mass size for each type was as follows: benign (24 mm), high-risk (27.5 mm), and malignant (26 mm). The results regarding the overall analysis are presented in Table 1. In addition, sensitivities for different lesion subtypes for large and small breasts are given in Table 2 and Table 3, respectively. There were no negative reports from the patients regarding the comfortability of SAFE scanning procedure.

Another sensitivity analysis was performed regarding the breast densities of the patients. Since some patients were scanned only with ultrasonography, density information was only available for 70 patients. Breast densities were categorized as follows: A—fatty breast, B—normal fibroglandular tissue, C—heterogeneously dense, and D—dense. The sensitivities achieved were 86%, 75%, 65%, and 48%, respectively. The results are presented in Table 4.

**Figure 2:**
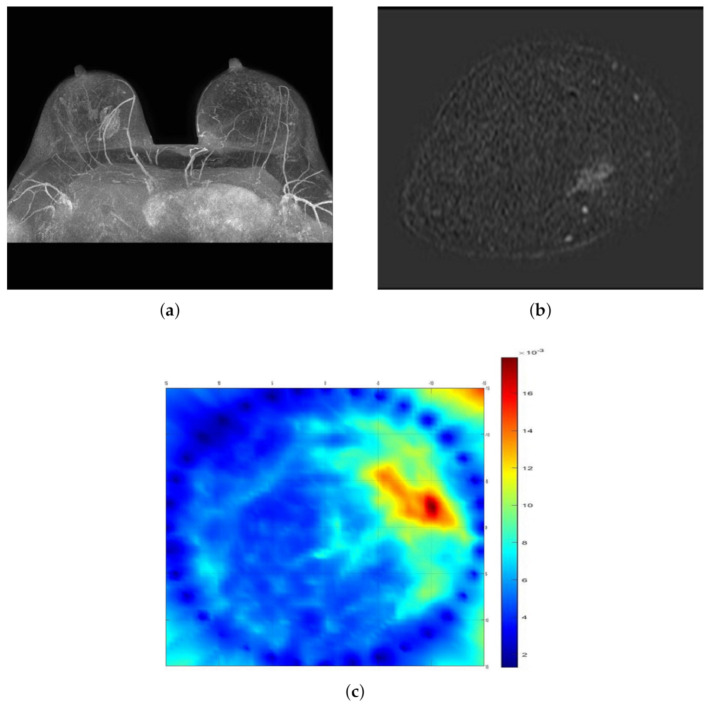
Comparison of SAFE lesion detection with clinical MRI: (**a**,**b**) MRI scans showing benign lesion in the right breast (axial and coronal planes), and (**c**) SAFE scan showing the concordance with MRI (coronal plane).

**Figure 3:**
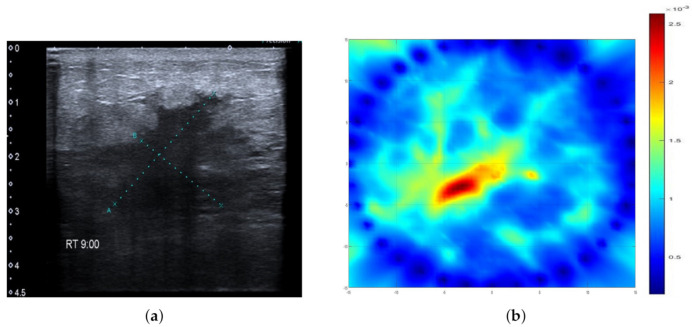
Comparison of SAFE lesion detection with clinical ultrasound: (**a**) ultrasound scan showing malignant lesion in the right breast and (**b**) SAFE scan showing the concordance with ultrasonography.

**Figure 4:**
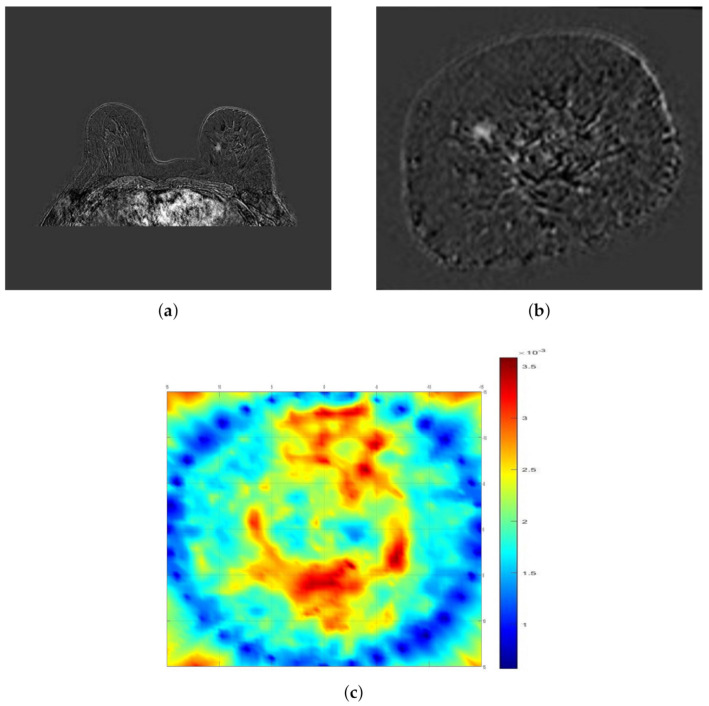
Comparison of SAFE image with clinical MRI: (**a**,**b**) MRI scans showing malignant lesion in the left breast (axial and coronal planes) and (**c**) SAFE scan showing no concordance with MRI (coronal plane) because of air gaps.

**Figure 5:**
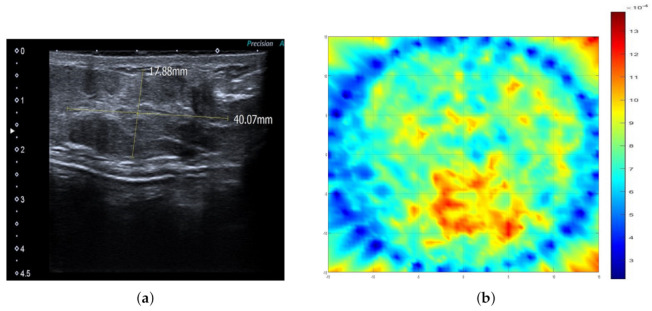
Comparison of SAFE image with clinical ultrasound: (**a**) ultrasound scan showing benign lesion in the left breast and (**b**) SAFE scan showing no concordance with ultrasonography because of air gaps.

Example cases where SAFE correctly detected and localized benign and malignant breast lesions together with the corresponding MR and ultrasound scans are given in Figure 2 and Figure 3, respectively. In both cases, the involved patients had large breasts. On the other hand, SAFE images of patients with small breasts are presented in Figure 4 and Figure 5. Conventional imaging detected malignant and benign lesions, whereas the lesions could not be detected using the SAFE device. Correctly detected cases (72 in total) were characterized by high-intensity localized abnormalities (Figure 2c and Figure 3b), which were concordant with clinically reported position and size of the lesion. Additionally, the approximated shape of the detected abnormality was concordant with the shape of the lesion that was detected by radiological imaging. Contrary to correctly detected cases, undetected ones (43 in total) were characterized by a random distribution of high-intensity abnormalities (Figure 4c and Figure 5b), with no concordance to the reported position and size of the lesion. The maximum average intensity for benign and malignant detected cases was 0.013 and 0.021, respectively. For undetected cases, maximum average intensity values were 0.008 for benign and 0.007 for malignant cases.

## 4. Discussion

A MWI-based breast cancer screening and early diagnosis prototype was developed for a broad range frequency breast examination. Patient discomfort during the scanning procedure was not reported as the device itself does not require breast compression for scanning. The time needed for each examination session was comparable to mammography, as bilateral scanning required approximately 20 min in total per patient. If the imperfection of the device design (incompatibility of the small breast with intended matching cups) is neglected, a correct detection rate of 74% was achieved. This level of sensitivity is similar to the one reported by Alan W. Preece [16] and is in the range of mammography sensitivity in the diagnostic settings, which may range from 45% in dense breasts up to the 100% in fatty ones [34]. The performance of SAFE was quite high in lower density breasts (type A and B), with sensitivities of 86% and 75%, respectively. On the other hand, SAFE performance decreased notably in dense breasts, with a sensitivity of 48%. This sudden drop in sensitivity must be further investigated in larger patient groups, given that 90% of undetected cases in dense breasts were in patients with small breasts. We believe that the production of new and more compatible matching cups will certainly increase the overall sensitivity of 63% and specifically the sensitivity in dense breasts, as the presence of air gaps and their effect on the device accuracy will be minimized. Together with effective detection, the use of non-ionizing radiation gives an advantage to the SAFE device over mammography, especially for younger women. At the current stage of development, SAFE can provide only 2-D images in the coronal plane. This means that information about the lesion location in the antero-posterior direction cannot be obtained. Such a limitation can be overcome by further enhancing the device configuration and imaging algorithm used. Since SAFE detects both benign and malignant lesions, its potential to differentiate between the two should be further investigated. SAFE pseudo color images indicated that the maximum average of a colormap intensity for malignant cases (0.021) was approximately 62% greater than in benign cases (0.013). Considering the fact that colormap intensity values are greatly affected by breast volume, breast-cup compatibility, and lesion vertical location, further studies need to be conducted so that the lesion classification can be done based only on SAFE images.

## 5. Conclusions

Microwave imaging represents an emerging technology in screening and early diagnosis of breast cancer. Its appealing features such as the use of non-ionizing electromagnetic radiation and absence of breast compression together with cost-effectiveness make it an attractive routine diagnostic tool that can be used for repeated scanning of the same patient, a feature that mammography cannot provide. These advantages make SAFE particularly appealing for younger women, especially women with hereditary genetic mutations, who are at considerable risk of developing breast cancer, and who have to start screening from an early age. Even though this is only a preliminary study, in terms of patients studied, we report one of the most comprehensive clinical assessments of MWI to date. However, the number of patients involved is not sufficient to derive a definite conclusion. Further clinical trials are planned in order to address all the issues discussed in this study and to provide clearer insight into the SAFE potential clinical role.

## Figures and Tables

**Table 1:** Patient sensitivity scores.

	All	Small Breast	Large Breast	Benign	High-Risk	Malignant
Patient No.	115	55	60	66	8	41
Sensitivity	72(63%)	28(51%)	44(74%)	42(64%)	7(88%)	24(59%)

Note: Sensitivity score of SAFE device based on the comparison with other imaging modalities used for screening and detection (Ultrasound, Mammography and MRI). An overall sensitivity of 63% was achieved, where sensitivity was lower in smaller breasts.

**Table 2:** Large Breast Analysis.

	All	Benign	High-Risk	Malignant
Patient No.	60	29	6	25
Sensitivity	44(74%)	22(76%)	5(84%)	17(68%)

Note: Sensitivity of SAFE device based on the comparison with other imaging modalities used for screening and detection (Ultrasonography, Mammography and MRI) in patients with large breast.

**Table 3:** Small Breast Analysis.

	All	Benign	High-Risk	Malignant
Patient No.	55	38	2	15
Sensitivity	28(51%)	20(53%)	2(100%)	6(40%)

Note: Sensitivity of SAFE device based on the comparison with other imaging modalities used for screening and detection (Ultrasonography, Mammography and MRI) in patients with small breast.

**Table 4:** Density Analysis.

	All	A	B	C	D
Patient No.	70	7	27	17	19
Sensitivity	46(66%)	6(86%)	20(75%)	11(65%)	9(48%)

Note: Sensitivity of SAFE device based on the breast density. An overall sensitivity of 66% was achieved for this group of patients, where the performance of SAFE decreases with increased breast density.

## Data Availability

The data are not publicly available due to Governmental and Institutional (Marmara University Hospital Breast Center) regulations and non-violation of patients privacy.
